# Draft Genome Sequences for Canadian Isolates of Pectobacterium carotovorum subsp. brasiliense with Weak Virulence on Potato

**DOI:** 10.1128/genomeA.00240-15

**Published:** 2015-04-09

**Authors:** Xiang (Sean) Li, Kat (Xiaoli) Yuan, Jeff Cullis, C. André Lévesque, Wen Chen, Christopher T. Lewis, Solke H. De Boer

**Affiliations:** a Canadian Food Inspection Agency, Charlottetown Laboratory (CFIA-CL), Charlottetown, Prince Edward Island, Canada; b Agriculture and Agri-Food Canada (AAFC), Ottawa, Ontario, Canada

## Abstract

Pectobacterium carotovurum subsp. brasiliense causes soft rot and blackleg diseases on potato. Here, we report the draft genome sequences of three weakly virulent P. carotovurum subsp. brasiliense strains isolated in Canada. Analysis of these genome sequences will help to pinpoint differences in virulence among P. carotovurum subsp. brasiliense strains from tropical/subtropical and temperate regions, such as Canada and United States. A small number of key factors for adaptation to this bacterium's specific environmental niche were also evaluated.

## GENOME ANNOUNCEMENT

Blackleg is an important disease of potato that causes significant losses to potato crops not only in the field but also in storage. In the past, the causal agent of the potato blackleg disease was found to be Pectobacterium atrosepticum. Recent studies indicated that other pectolytic bacteria also cause the blackleg disease in various potato-growing regions, for example, Pectobacterium wasabiae (1) and Pectobacterium caratovorum (2) in some temperate regions, Dickeya solani in European countries (3), and P. carotovorum subsp. brasiliense in Brazil (4) and some African countries (5).

P. carotovorum subsp. brasiliense was considered the only causal agent of potato blackleg in Brazil after an extensive survey in the Brazilian state of Rio Grande Do Sul (6), and was a major cause of potato blackleg in South Africa (5). More recently, P. carotovurum subsp. brasiliense was also found in temperate regions such as the United States (7), Canada (1), and Israel (8). In particular, De Boer et al. (1) found that Canadian isolates of P. carotovurum subsp. brasiliense were clearly less virulent than Brazilian strains in both greenhouse and field conditions. Whether or not these differences in aggressiveness could be assigned to specific genomic differences was a major trigger for this sequencing project. Comparative genomics data would provide important insight into genomic differences that differentiate the highly virulent tropical strains from temperate isolates of P. carotovurum subsp. brasiliense.

In this study, three Canadian strains (CFIA1001, 1009, and 1033) isolated from blackleg-infected potato stems were decoded using paired-end Illumina HiSeq sequencing technology with TrueSeq version 3 chemistry (National Research Council Canada, Saskatoon, Saskatchewan, Canada). In total, 2,120,303,100 bp, 1,344,825,088 bp, and 4,059,728,734 bp were obtained from 300-bp inserts to provide approximately 27×, 21×, and 37× genome coverages for strains CFIA1001, CFIA1009, and CFIA1033, respectively. After quality checking and initial *de novo* assembly using the Velvet assembler (9), the draft genome sizes for these three strains are as follows. CFIA1001 is 4,764,478 bp comprising 28 contigs with 52.3% G+C content; CFIA1009 is 4,756,221 bp comprising 43 contigs with 51.3.4% G+C content; and CFIA1033 is 4,701,524 bp comprising 79 contigs with 51.4% G+C content. Annotations were conducted on the RAST server using the Glimmer 3 option (10) and it predicted 4,457, 4,442, and 4,471 protein-coding genes, including 85, 81, and 77 noncoding RNA genes for CFIA1001, CFIA1009, and CFIA1033, respectively. A number of predicted virulence-related factors, phage-related loci, motility, and chemotactic genes were identified in the genome, which may facilitate its specific pathogenicity in specific environments.

Further analysis of these strains will especially focus on environmental niche-adapted features and pathogenicity-related determinants to provide detailed insight into the genetics of ecological adaptation, virulence, and plant-pest interactions of this widely distributed pathogen.

### Nucleotide sequence accession numbers.

The draft genome sequences of P. carotovorum subsp. brasiliense strains CFIA1001, CFIA1009, and CFIA1033 have been deposited in GenBank under the accession numbers JPSM00000000, JPSN00000000, and JPSO00000000, respectively. The versions described in this paper are the first versions.
